# Editorial: Reading and writing the neural code for neuroprosthetics

**DOI:** 10.3389/fnins.2023.1130921

**Published:** 2023-02-06

**Authors:** Alberto Mazzoni, Michela Chiappalone

**Affiliations:** ^1^The Biorobotics Institute, Sant'Anna School of Advanced Studies, Pisa, Italy; ^2^Department of Informatics, Bioengineering, Robotics and Systems Engineering (DIBRIS), University of Genova, Genova, Italy

**Keywords:** neurostimulation, recording, electrophysiology, decoding, modeling

Disorders and trauma of the nervous system, either at the central or peripheral level, have profound disabling effects on patients, severely affecting their quality of life. Restoring the physiological function of the dysfunctional nervous system is then a primary mission for the scientific and medical community.

Recent studies are exploring advanced electroceutical approaches, which can represent a promising alternative to standard therapies. In particular, major progresses in the fields of bioelectronics and neural engineering have led to the development of efficient upper and lower limb neuroprostheses replacing the function of missing/non-functional limbs. Neuroprostheses rely on brain machine interfaces both to decode motor intentions and to provide sensory feedbacks. Hence, in order to develop a successful neuroprosthesis, it is essential to understand how to interface it with the nervous system, both in terms of hardware (electrodes, sensors) and in terms of being able to decode the state of the system from acquired signal and to design the most efficient stimulation to reach the desired outcome. This latter aspect, i.e., the ability to “read” and/or “write” the neural code is the subject of this Research Topic.

In this issue, Wimmer et al. elicited a wrist movement through neuromuscular electrical stimulation and then simultaneously measured spinal cord potentials (SCPs) and somatosensory evoked potentials (SEPs) in the EEG. This enabled them to quantify the strength and the directionality of the information flow from spine to brain (and vice versa) only by means of non-invasive recordings. A clear understanding of the information flow from periphery to the cortex is a key issue in neuroprosthetics (Shokur et al., 2021).

Saldanha et al. analyzed the impact of electrode-site placement on long-term intracortical microstimulation (ICMS) delivered to chronically implanted rats. They used custom-made single-shank silicon microelectrode array with sites on the edge, center, and tip and evaluated the role of electrode-site placement in terms of ICMS detection thresholds and voltage transients. They found that electrodes on the tip of the device outperformed those on both the edge and center in terms of the effect per charge delivered. During long-term evaluations, the tip and edge sites consistently elicited behavioral response with less charge, compared to center sites. Thus the authors demonstrated the important of electrode-site placement for ensuring long-term stability of intracortical micro-stimulation, especially in the framework of neuroprosthetics applications.

On the reading side, current recording techniques provide neuroscientists and neuroengineers an unprecedented amount of online data from large populations of neurons, both in the brain and at the periphery. A key element for the optimal decoding of such populations is dimensionality reduction through the assessment of the interactions among neurons, which is the problem addressed by Mitskopoulos et al. in their work in this issue. While most of the theoretical tools employed to study interactions in neural activity are limited by assumptions, e.g., Gaussian distribution of the variables and interactions limited to the second order, the flow-based estimation of vine copulas proposed by the authors does not rely on any specific interaction model and is suited for heavy tailed distributions as those often present in neuroscience. In the work, the relevance of the high order interactions captured by the approach is shown in the neural responses recorded in mouse V1 while the animal is navigating in a virtual reality environment. Such population decoding techniques could be used to decode motor intention from the motor cortex of tetraplegic subjects to control neuroprostheses.

The reading process is also a key element for the realization of prosthetic devices characterized by a more natural control. This is the main focus of the work of Li et al., which provides a comprehensive overview on the applicability of Deep Learning (DL) techniques to improve the accuracy of surface electromyography (sEMG) pattern recognition to interpret hand gesture. The paper reviews the key techniques of DL-based sEMG pattern recognition for the prosthetic hand, including signal acquisition, signal preprocessing, feature extraction, classification of patterns, post-processing, and performance evaluation. Interestingly the paper also identifies possible challenges and limitations of the technique, mostly related to the high variability of sEMG signals and the limitation of appropriate hardware resources, which currently impede the exploitation of DL-based classification for real-time applications.

Finally, Bouton et al., in their contribution to this Research Topic, provide the first demonstration of decoding sulcal and subcortical activity related to both movement and tactile sensation in the human hand by using stereoelectroencephalography (SEEG) electrodes. Minimally invasive techniques, such as stereoelectroencephalography (SEEG) have become more widely used in clinical applications in epilepsy patients since they can lead to fewer complications, but they have not been quite used in the BCI field. To overcome this limitation, they propose this reading modality and compared its decoding performances with those obtained by electrocorticography electrodes (ECoG). Finally, they adopted deep learning methods to automatically classify various motor and sensory events for individual fingers with high accuracy. The paper by Bouton et al. opens up new avenues for a new class of minimally invasive brain-computer interface systems.

To conclude, the scientific community should pay more attention to determine “what” is useful to read and “what” is useful to write in the nervous system, in addition to “how” to read and “how” to write. The contributions to this Research Topic provide a step forward toward this direction, as it can be a real game changer to improve the functionality and the usability of the neuroprosthetic devices of the future.

## Author contributions

Both authors listed have made a substantial, direct, and intellectual contribution to the work and approved it for publication.
